# Development and validation of a 4-gene combination for the prognostication in lung adenocarcinoma patients

**DOI:** 10.7150/jca.37003

**Published:** 2020-01-29

**Authors:** Xiao-Hong Yin, Li-Ping Yu, Xiao-Hong Zhao, Qin-Mei Li, Xiao-Ping Liu, Li He

**Affiliations:** 1 Department of Hematology, Zhongnan Hospital of Wuhan University, Wuhan, Hubei province, China; 2 Wuhan University School of Health Sciences, Wuhan, Hubei province, China; 3 Department of Epidemiology, Department of Epidemiology, School of Health Sciences, Wuhan University, Wuhan, Hubei, China; 4 Department of Urology, Zhongnan Hospital of Wuhan University, Wuhan, Hubei province, China

**Keywords:** lung adenocarcinoma, prognostication, least absolute shrinkage and selection operator, survival analysis

## Abstract

**Objective:** To identify a multi-gene prognostic factor in patients with lung adenocarcinoma (LUAD).

**Materials and methods** Prognosis-related genes were screened in the TCGA-LUAD cohort. Then, patients in this cohort were randomly separated into training set and test set. Least absolute shrinkage and selection operator (LASSO) regression was performed to the penalized the Cox proportional hazards regression (CPH) model on the training set, and a prognostication combination based on the result of LASSO analysis was developed. By performing Kaplan-Meier curve analysis, univariate and multivariable CPH model on the overall survival (OS) as well as recurrence free survival (RFS), the prognostication performance of the multigene combination were evaluated. Moreover, we constructed a nomogram and performed decision curve analysis to evaluate the clinical application of the multigene combination.

**Results** We obtained 99 prognosis-related genes and screened out a 4-gene combination (including CIDEC, ZFP3, DKK1, and USP4) according to the LASSO analysis. The results of survival analyses suggested that patients in the 4-gene combination low-risk group had better OS and RFS than those in the 4-gene combination high-risk group. The 4-gene mentioned was demonstrated to be independent prognostic factor of patients with LUAD in the training set(OS, HR=11.962, P<0.001; RFS, HR=9.281, P<0.001) and test set (OS, HR=5.377, P=0.003; RFS, HR=2.949, P=0.104). More importantly, its prognosis performance was well in the validation set (OS, HR=0.955, P=0.002; RFS, HR=1.042, P<0.001).

**Conclusions** We introduced a 4-gene combination which could predict the survival of LUAD patients and might be an independent prognostic factor in LUAD.

## Introduction

Lung cancer represents the lion's share of cancer morbidity and mortality worldwide, with 2.1 million new lung cancer cases and 1.8 million deaths in 2018, or nearly 1 in 5 (18.4%) of the total cancer mortality^1^ in other words. Lung cancer is mainly classified into small cell lung cancer and non-small cell lung cancer. Approximately, 85% of patients are non-small cell lung cancer, of which lung adenocarcinoma (LUAD) and lung squamous cell carcinoma (LUSC) are the most common subtypes. According to the epidemiological surveys in recent years, the incidence of LUAD has exceeded that of LUSC in both smokers and non-smokers in many countries, accounting for almost a half of all lung cancers; Even worse, in some countries, the incidence of LUAD in women is multiplying, and it also becomes the most common pathological type of lung cancer in young people 2, 3. Worst of all, LUAD is a kind of non-small cell lung cancer with extremely high lethality in its advanced stage but no obvious symptoms in its early stages, and thus most patients with LUAD may have delayed detection due to the late onset of symptoms and lack of specificity^4^, ^5^. Thanks to the continuous improvements of novel treatments and diagnosis techniques^6^, the management strategies for LUAD have been improved considerably^7^, ^8^. However, the prognosis of LUAD patients is generally remaining to be unsatisfactory (the 5-year survival remains about 15%). Therefore, early diagnosis and treatment are crucial to the improvement of the prognosis of LUAD patients, which stresses the demands to develop biomarkers which can help identifying at-risk patients in that they can benefit from early interventions^9^. Biomarkers can be used for screening, diagnosis, and prognosis of tumors, but the individual biomarker is far from enough to meet clinical requirements^10^, ^11^. In consequence, combined detection of multiple biomarkers is advocated in clinical practice to improve sensitivity and specificity of diagnosis. Nowadays, biomarkers for lung cancer detection mainly include carbohydrate antigens, tumor embryo antigens, differentiation antigens, and proliferating antigens^12^, ^13^. Nevertheless, the prognostic performance of established biomarkers remains controversial and limited. Thus, in this study, we identified a 4-gene combination to predict the prognosis of patients with LUAD.

## Materials and methods

### Discovery datasets

The cancer genomic atlas (TCGA) program, hosted by the National Cancer Institute's (NCI), molecularly studied more than 20,000 primary cancers and corresponding normal samples across 33 cancer types and the genomic data of these 33 cancer types was uniformly reposited in the NCI's Genomic Data Commons (GDC), which facilitates precision medicine. The expression profile of TCGA-LUAD cohort was measured by using the Illumina HiSeq 2000 RNA Sequencing platform created by the University of North Carolina TCGA genome characterization center. Apart from that, the gene expression profile is generated using GDC mRNA quantification analysis pipeline and obtained from UCSC Xena (https://xenabrowser.net/datapages/). The matched clinical data was also obtained from GDC Xena Hub (https://gdc.xenahubs.net). Patients in the TCGA-LUAD including 503 LUAD samples were included in the presents according to the following inclusion criteria: patients with primary lung adenocarcinoma; patients whose survival information was documented; patients whose tumor tissues were subjected to RNA sequencing; patients who were not previously treated; Meanwhile, patients meeting the exclusion criteria (patients with history of other malignancies, patients whose tumor tissues were not undertaken to RNA sequencing analysis, patients with metastasis disease; patients whose survival information was not available; repeated mRNAs sequencing samples) were excluded. Then, the remaining patients in the TCGA-LUAD were randomly assigned to a training set and test set in a 1:1 ratio for subsequent analysis using the R package “caret”.

### Validation dataset

To perform an independently external validation, we used GSE31210 as a validation dataset. The gene expression profile and clinical data were downloaded from the Gene Expression Omnibus (GEO)(https://www.ncbi.nlm.nih.gov/geo/). GSE31210 was measured by Affymetrix Human Genome U133 Plus 2.0 Array including 226 LUAD samples^11^, ^14^. The inclusion and exclusion criteria were the same with those used in the TCGA-LUAD.

### Statistical analysis

Firstly, we screened the prognosis-related genes in TCGA-LUAD by using univariate Cox proportional hazards regression (CPH) model, genes with Bonferroni corrected P <0.05 were obtained. Then, R package “caret” was implied to perform stratified random sampling based on survival status, which was randomly divided into training set and test set according the ratio of 1:1. The R package “glmnet” was used to perform a least absolute shrinkage and selection operator (LASSO) regression to penalized CPH model for minimizing the overfitting of the Cox regression on the training set. After that, the risk score of each patient was calculated utilizing the predict function based on this penalized CPH model in the training set, test set and validation set. Since time-dependent survival receiver operating characteristic (ROC) is an important method to evaluate the predictive ability of prognostic models, R packages “survivalROC” was utilized to perform time-dependent ROC for identifying optimal cutoff value corresponding to the highest true-positive rate and the lowest false-positive rate, which was then used to divide the LUAD patients into low-risk groups and high-risk groups. Subsequently, prognostic significance of the multigene combination was estimated by evaluating the differences of Kaplan-Meier survival curve analysis between the two groups in terms of overall survival (OS) and relapse-free survival (RFS). Furthermore, univariate and multivariable CPH models were utilized to identify independent prognostic factors associated with survival, and we considered risk score, age, gender, and pathologic stage as covariate. The above survival analysis was conducted using the R packages “survminer” and “survival”.

After obtaining the above results, GSE31210 was used as an external validation dataset. Therefore, the risk score of each patient was calculated based on the regression coefficients of genes in the multigene combination, and the cut off value was also found as introduced above. According to the optimal cutoff value, the LUAD patients in the external validation set were separated into high-risk group and low-risk group. In addition, Kaplan-Meier survival curve analysis, univariate and multivariable CPH model were performed to validate the prognostic performance of discovery dataset. To further confirm the clinical relevance of the multigene combination, we compared the prognostication performance of the multigene combination with several well-established biomarkers^15^-^26^ in terms of C-index. All statistical analyses were performed using the R 3.5.2 software, and P<0.05 was considered statistically significant.

### Enrichment analysis

In order to explore the potential molecular mechanisms of the prognosis-related genes affecting the survival of LUAD patients, Gene Ontology (GO) and Kyoto Encyclopedia of Genes and Genomes (KEGG) pathway enrichment analysis were conducted by using the DAVID online tool (https://david.ncifcrf.gov/)^27^, ^28^. We considered P<0.05 and false discovery rate (FDR) P<0.05 as significantly enrichment, and used R package “ggplot2” to visualize the significantly enriched GO and KEGG terms.

### Clinical application of the 4-gene combination

Nomogram is a commonly used prediction tool in medicine. It integrates diverse prognostic and determinant variables to generate individual and numerical probabilities of clinical events^29^. To clarify the clinical application ability of the 4-gene combination, we built a 4-gene combination based nomogram estimating the 3-year, and 5-year OS of LUAD patients, which included the age, gender, stage, and the risk score of each BC patient into a multivariate survival model. After using 1000 resampled bootstraps to internally verify the predicted values of the nomogram, we applied the R package "rms" to draw the nomogram. Meantime, we performed decision curve analysis (DCA) to verify the clinical role of the nomogram for the 4-gene combination^30^.

## Results

### Characteristics of LUAD patients in the discovery dataset and validation dataset

A total of 729 LUAD patients with survival information were included in the present study. There were 502 patients in the discovery dataset, and the characteristics of patients in the training set and test set are balanced (median age in years was 66 [range 38-87], 138 females [52.98%], and 118 males [47.01%] in the training set; median age in years was 67 [range 40-88], 133 females [52.99%] in the test set). Meanwhile, there were 226 patients in the validation dataset (GSE31210) (median age in years was 61 [range 30-76], 121 females [53.54%], and 105 males [46.46%]), detailed characteristics of patients in the training set, test set, and GSE31210 were summarized in supplementary table 1.

### Development of the prognostic multigene combination and validation of its prognostic performance

**Figure 1:**
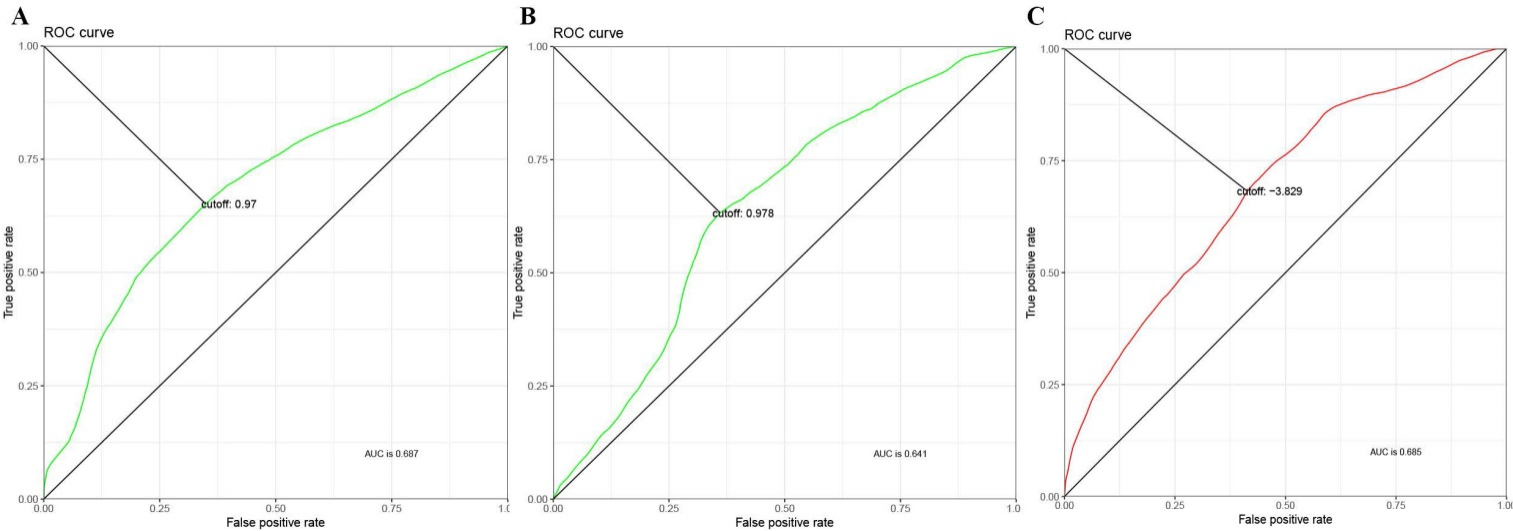
Time-Dependent ROC Curve of 4-gene combination. (A) ROC in the training set. (B) ROC in the test set. (C) ROC in the validation set.

**Figure 2:**
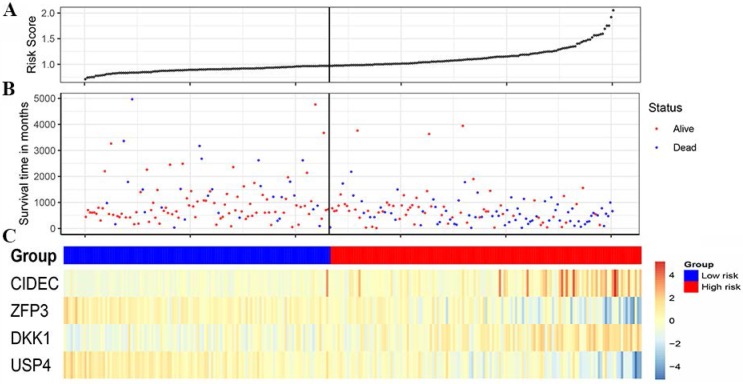
Characteristics of the 4-gene combination of the discovery cohort. (A) Risk score (On the left is the low-risk group and on the right is the high-risk group). (B) Survival time in days (Red dot indicates Alive, blue dot indicates death). (C) Gene expression heatmap (The blue color is the low-risk group and the red color is the high-risk group)

A total of 99 prognosis-related genes were identified in the TCGA-LUAD after Bonferroni correction(Supplementary table 2). As a result of LASSO penalized CPH model in the training set, four genes (CIDEC [ubiquitin specific peptidase 4], ZFP3 [ZFP3 zinc finger protein], DKK1 [dickkopf WNT signaling pathway inhibitor 1], and USP4 [ubiquitin specific peptidase 4]) were finally identified. Thus, a 4-gene combination was built on the basis of the coefficients of these 4 genes in the LASSO penalized CPH model (supplementary table 3). According to the optimal cutoff value of 0.97, 0.978 and -3.829 (Figure 1), we classified the patients in training set, test set and validation set into the 4-gene combination high-risk group and 4-gene combination low-risk group, respectively. The characteristics of the 4-gene combination were shown in Figure 2.

**Figure 3:**
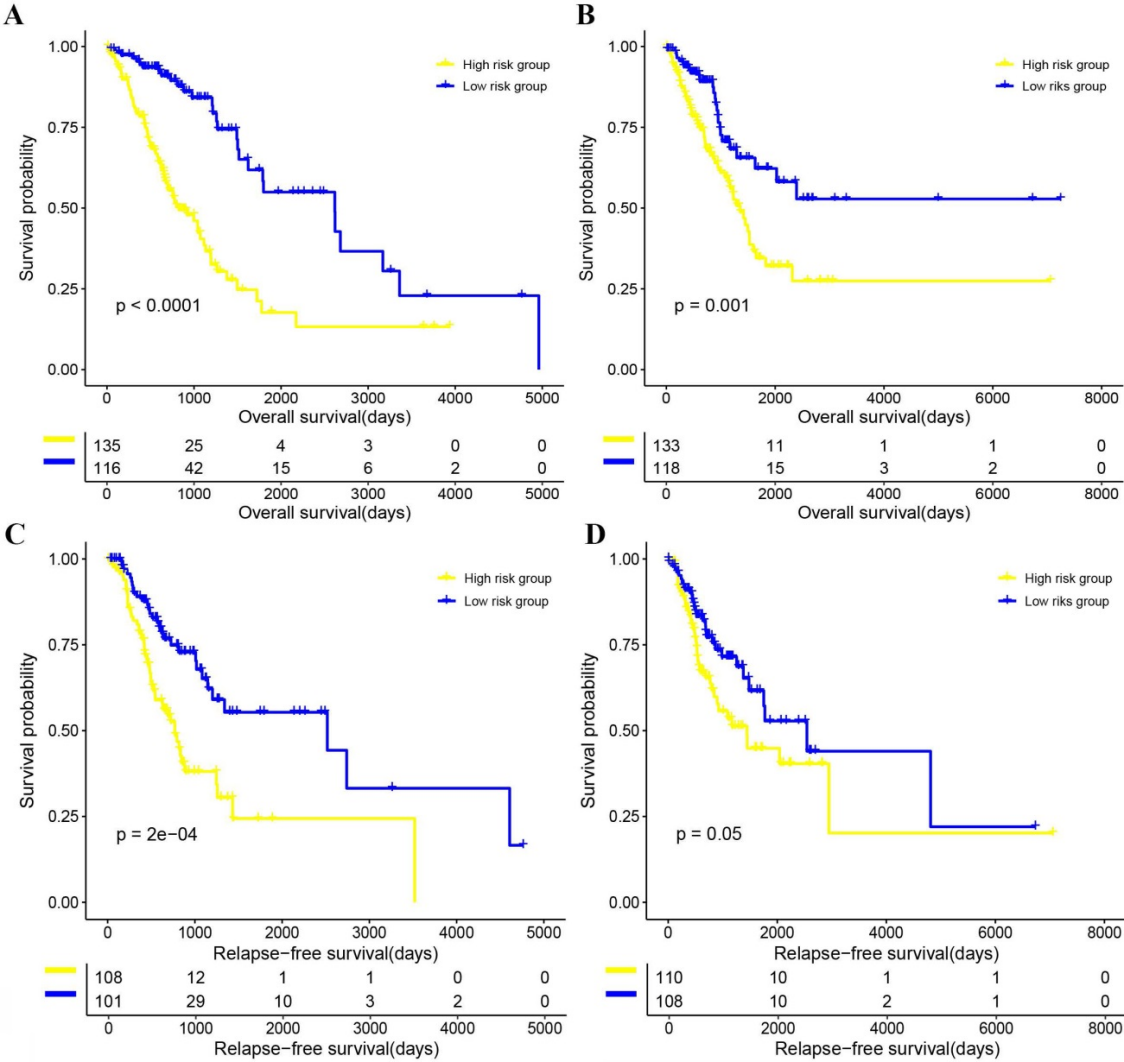
The correlations between the 4-gene combination and the overall survival (OS) and relapse-free survival (RFS) of patients with LUAD. (A) OS in the training set. (B) OS in the test set. (C) RFS in the training set. (D) RFS in the test set.

**Figure 4:**
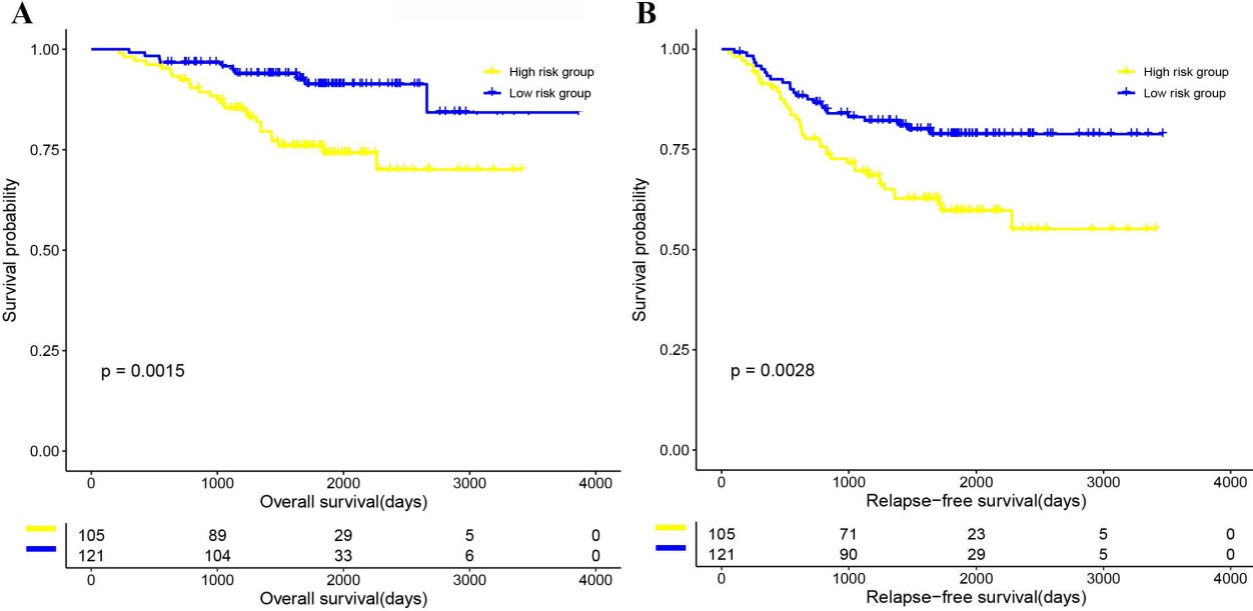
Kaplan-Meier curve survival analysis on validation set. (A) OS in the validation set. (B) RFS in the validation set.

The Kaplan-Meier curve of OS and RFS, and univariate CPH model of OS and RFS results suggested that patients in the 4-gene combination low-risk group was associated with better OS and RFS compared with those in the 4-gene combination high-risk group in the training set (OS, hazards ratio (HR)=11.962, 95% CI: 6.232-22.961, P<0.001; RFS, HR=9.281, 95% CI: 4.064-21.193, P<0.001; figure 3 and supplementary table 4), test set (OS, HR=5.377, 95% CI: 1.736-16.657, P=0.003; RFS, HR=2.949, 95% CI: 0.800-10.867, P=0.104; figure 3 and supplementary table 4) and validation set (OS, HR=1.057, 95% CI: 1.016-1.079, P=0.002; RFS, HR=1.042, 95% CI: 1.018-1.066, *P*<0.001; figure 4 and Supplementary table 4). Meanwhile, the multivariable CPH model of OS and RFS results exhibited that the 4-gene combination might be an independent prognostic factor of LUAD patients in the training set, test set, and validation set (Supplementary table 4) after adjusting other clinical factors such as age, gender, smoking status, and stage. The results showed that the 4-gene combination was effective in predicting OS and RFS in LUAD patients.

### Validation of the prognostic performance of the 4-gene combination

As shown in figure 3, the Kaplan-Meier curve of OS and RFS, and univariate CPH model of OS and RFS (OS, HR=0.955, 95% CI: 1.016-1.079, P=0.002; RFS, (HR)=1.042, 95% CI: 1.018-1.066, P<0.001; Figure 3 and Supplementary table 4) in the 4-gene combination low-risk group were also significantly better than those in the 4-gene combination high-risk group. In addition, although the multivariable CPH model of OS and RFS results were not statistically significant (OS, HR=1.029, 95% CI: 0.997-1.062, P=0.076; RFS, (HR)=1.024, 95% CI: 0.999-1.049, P=0.052; Supplementary table 4), it indicated that the 4-gene combination has a tendency of being an independent prognostic factor in the validation cohort. The above results suggested that the prognostic performance of the 4-gene combination in the discovery dataset and the validation dataset was significant.

Given the fact that several well-established prognostic signatures or biomarkers have been introduced, we tried to compare the prognostic performance of these biomarkers with our 4-gene signature. Zhang et al. introduced a 3-gene signature based on the main members of kinesin family member genes (KIF14, KIF18B, and KIF20A), and they demonstrated that it can significantly stratify patients into low-risk group and high-risk group^15^. Sun et al. suggested a 2-gene signature composed of BRCA1 and ERBB3 and applied it to the prognosis prediction in patients with lung adenocarcinoma^16^. Meanwhile, there were several well-established biomarkers including (ALDH1A1^17^, CD117^18^, CELIAC1^19^, CX3CL1^20^, IFITM1^21^, ITGA2B^22^, LETM1^23^, PHLPP2^24^, RRBP1^25^, and TMEM213^26^) in lung adenocarcinoma. We calculated the C-indexes of our 4-gene signature and the well-established signatures mentioned above. As shown in the following figure 1, the C-index of the 4-gene signature was obviously higher than that of the other biomarkers in the training set, test set, and the independent validation set GSE31210, indicating that our 4-gene signature has superior predictive performance compared to other models and is more reliable in clinical settings.

### The results of the enrichment analysis

**Figure 5:**
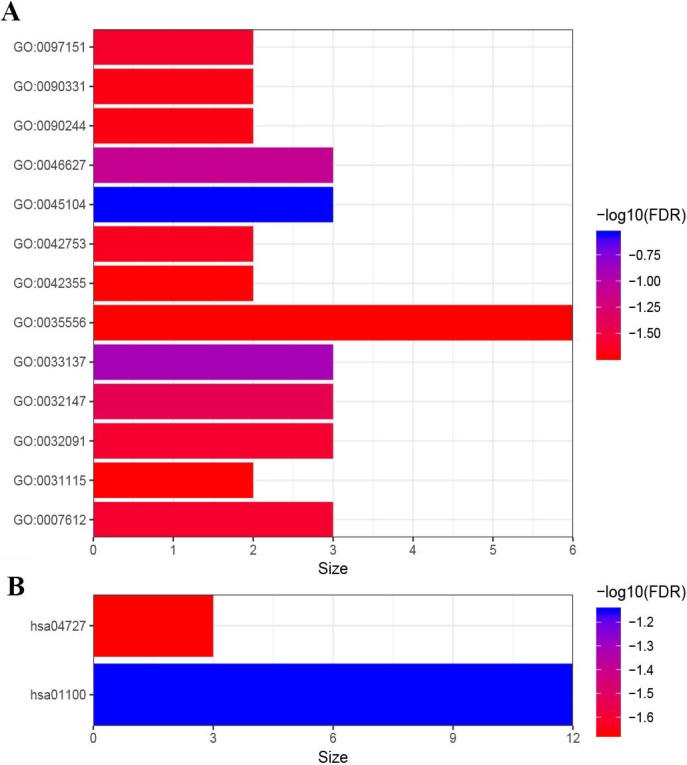
Functional enrichment analysis of 99 genes. (A) GO enrichment analysis. (B) KEGG enrichment analysis. GO:0045104 intermediate filament cytoskeleton organization; GO:0033137∼negative regulation of peptidyl-serine phosphorylation; GO:0046627∼negative regulation of insulin receptor signaling pathway; GO:0032147∼activation of protein kinase activity; GO:0032091∼negative regulation of protein binding; GO:0007612∼learning; GO:0097151∼positive regulation of inhibitory postsynaptic potential; GO:0042753∼positive regulation of circadian rhythm; GO:0090244∼Wnt signaling pathway involved in somitogenesis; GO:0090331∼negative regulation of platelet aggregation; GO:0031115∼negative regulation of microtubule polymerization; GO:0042355∼L-fucose catabolic process; GO:0035556∼intracellular signal transduction. hsa01100∼Metabolic pathways; hsa04727∼GABAergic synapse.

In order to have a rudimentary knowledge of the biological meaning of the prognosis-related genes, GO and KEGG enrichment analysis were conducted. As shown in Figure 5, the prognosis-related genes were mostly enriched in GO terms related to gene regulation (“negative regulation of peptidyl-serine phosphorylation”, “negative regulation of insulin receptor signaling pathway”, “negative regulation of protein binding”, “positive regulation of inhibitory postsynaptic potential”, “positive regulation of circadian rhythm”, “negative regulation of platelet aggregation” and “negative regulation of microtubule polymerization”), cell proliferation (“intermediate filament cytoskeleton organization”, “activation of protein kinase activity” and “L-fucose catabolic process”) and signal transduction (“intracellular signal transduction” and “Wnt signaling pathway involved in somitogenesis”) (Figure 5). Furthermore, the results of KEGG pathway enrichment analysis of prognosis-related genes showed that these genes were mainly enriched in metabolic pathways (“Metabolic pathways” and “GABAergic synapse”) (Figure 5).

### Clinical application of the 4-gene combination

**Figure 6:**
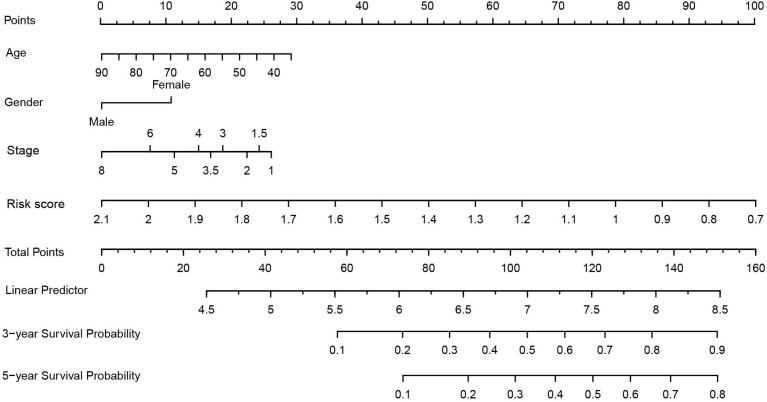
Nomogram construction based on 4-gene combination.

**Figure 7:**
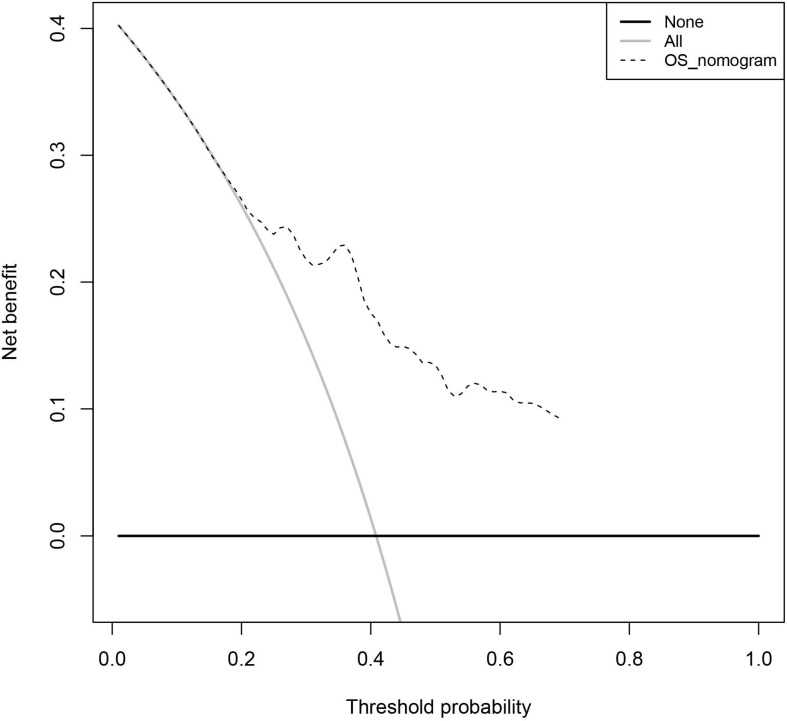
The decision curve analysis of the 4-gene combination.

As shown in figure 6, we constructed a prognostic nomogram which included age, gender, stage and the risk score to predict the 3- and 5-year OS of patients with LUAD. The internally and externally validated Harrell's c index were 0.726 and 0.654 , indicating that the 4 gene combination performed well in clinical application. DCA could determine a range of threshold probabilities for a prediction mode, as shown in Figure 7, the nomogram threshold probability based on 4-gene combinations was significantly better than the default strategies of treating all or none at a threshold probability more that 4%.

## Discussions

Given the low rate of 5-year survival as well as high rate of recurrence of LUAD patients, and also early diagnosis along with intervention can improve the clinical outcomes of LUAD patients, identification and evaluation of novel biomarkers for patients with LUAD is of great importance. In the present study, univariate CPH analysis was performed to analyze the relations between the expression of genes and the OS of patients with LUAD, and the results exhibited that 99 genes were associated with the OS of LUAD patients. LASSO (34) was introduced in order to improve the prediction accuracy and interpretability of regression models by forcing certain coefficients to be set to zero, and effectively to choose a simpler model that did not include those coefficients. Based on this, the above 99 genes were included into a LASSO penalized CPH model, and as a result 4 genes with none-zero coefficients in this model were obtained. Therefore, the risk score of each LUAD patients was calculated, and the 4-gene combination was built for prediction of overall survival and recurrence of patients with LUAD.

The OS and RFS of LUAD patients in the 4-gene combination low-risk group significantly superior compared with those in the 4-gene combination high-risk group, and the 4-gene combination was shown to be an independently prognostic combination in LUAD. Actually, studies have reported that these four genes in the combination are associated with cancer progression or suppression. DKK1 was a member of the dickkopf family and members of this family were secreted proteins characterized by two cysteine-rich domains that mediate protein-protein interactions^31^. This gene played a role in embryonic development and might be important in bone formation in adults^32^. Elevated expression of this gene had been observed in numerous human cancers and it corresponding protein promoted proliferation, invasion and growth in several kinds of cancer cell lines^33^, ^34^. Aufderklamm S et al. demonstrated that DKK-1 could inhibit osteoblast activity by blocking the Wnt pathway, which led to progression of metastatic prostate cancer^35^. CIDEC, a member of the cell death-inducing DNA fragmentation factor-like effector family, could promote lipid droplet formation in adipocytes and mediate adipocyte apoptosis^36^. It was reported that the CIDE family regulated lipid metabolism and played an important role in the development of metabolic disorders such as obesity^37^, insulin resistance^38^ and hepatic steatosis^39^. Ming Yu et al. found that CIDE family was highly expressed in clear cell renal cell carcinoma^40^, USP4, the protein encoded by this gene is a protease that deubiquitinates target proteins, shuttles between the nucleus and cytoplasm and is involved in maintaining operational fidelity in the endoplasmic reticulum^41^. Hou X et al. demonstrated that USP4 was significantly upregulated in head and neck squamous cell carcinoma. USP4 might negatively regulates RIP1-mediated NF-κB activation and promotes TNF-α-induced apoptosis in FaDu cells, as well as directly interacted with receptor-interacting protein 1 (RIP1) and deubiquitinated K63-linked ubiquitination from RIP1^42^. Yao R et al. suggested that esophageal cancer patients with high USP4 expression had relative longer survival time^43^. Some researchers also concluded that USP4 expression might be a favorable biomarker for OS and RFS in LUAD patients, while it was worth noting that USP4 expression has no prognostic value for OS and RFS in lung squamous cell carcinoma^44^. In addition, Ye et al. suggested that USP4 had anti-cancer effect in breast cancer, and found that USP4 could inhibited the growth of tumors in a mouse tumor xenograft model^45^. ZFP3 contains a conserved structural motif that mediates its binding to protein, DNA and RNA, in humans ZFP3 has been localized to chromosome 17p12-17pter^46^. Ming et al. reported that the ZFP3 had protective effect in clear cell renal cell carcinoma^47^, which was consistent with our research.

Meanwhile, the GSE31210 dataset was used to validate the prognostication ability of the 4-gene combination. It has been proved that 4-gene combination can be used as an independent prognostic factor for LUAD patients in the validation cohort. Results of functional enrichment analysis of the prognosis-related genes suggested that the prognosis-related genes were mainly enriched in gene regulation, cell proliferation and signal transduction related GO terms and pathways, this was in accordance with the prognostic value of the 4-gene combination. In addition, we obtained good results by detecting the clinical potency of the 4 gene combination by nomogram and DCA. Collectively, we suggested that our 4-gene combination was a good supplement for the prognosis prediction of patients with LUAD.

Although the four gene combinations showed excellent performance in the training set, test set and validation set, it had the following defects. First, this study was an integration and reanalysis of existing published LUAD gene expression studies. Although it showed good performance in prediction of the survival and recurrence of LUAD patients, it had not been verified by large scale prospective trials. Second, the associated mechanisms had not been validated in LUAD cells. Based on this, our follow-up researches will focus on verifying the conclusions of this study in terms of clinical application and molecular mechanisms.

In conclusions, we introduced a 4-gene combination which could predict the survival of LUAD patients and might be an independent prognostic factor in LUAD.

## Supplementary Material

Supplementary figures and tables.
