# Supplementary material for: Development and validation of a 4-gene combination for the prognostication in lung adenocarcinoma patients
Source: J Cancer. 2020 Jan 29;11(7):1940–8. doi: 10.7150/jca.37003 (PMC7052877; doi:10.7150/jca.37003)
Supplement: Supplementary file 1 — Supplementary figures and tables. [file jcav11p1940s1.pdf]

Supplementary table 1 Characteristics of patients in the training set, test set, and validation set.

	Characteristics	Low risk	High risk
Training set	Age (year)	66(43-83)	65(38-87)
	Gender		
	Male	45(38.79%)	68(50.37%)
	Female	71(61.21%)	67(49.63%)
	Pathological stage		
	I	75(64.66%)	55(40.74%)
	II	22(18.97%)	31(22.96%)
	III	12(10.34%)	29(21.48%)
	IV	0	8(5.93%)
	NA	7(6.03%)	12(8.89%)
Test set	Age (year)	66(41-88)	67(40-84)
	Gender		
	Male	53(44.92%)	65(48.87%)
	Female	65(55.08%)	68(51.13%)
	Pathological stage		
	I	69(58.47%)	65(48.87%)
	II	28(23.73%)	38(28.57%)
	III	12(10.17%)	17(12.78%)
	IV	5(4.24%)	10(7.52%)
	NA	4(3.39%)	3(2.26%)
GSE31210	Age (years)		
	<=61	70(57.85%)	52(49.52%)
	>61	51(42.15%)	53(50.48%)
	Gender		
	Female	69(57.02%)	52(49.52%)
	Male	52(42.98%)	53(50.48%)
	Smoking status		
	Ever-smoker	54(44.63%)	57(54.29%)
	Never-smoker	67(55.37%)	48(45.71%)
	Pathological stage		
	IA	70(57.85%)	44(41.9%)
	IB	27(22.31%)	27(25.71%)
	II	24(19.83%)	34(32.38%)

Supplementary table 2. The 99 genes associated with prognosis.

**Gene**	**Coefficient**	**HR**	**LCI**	**UCI**	***P* value**	**FDR**
ZNF709	(0.364)	0.695	0.602	0.802	*<0.001*	0.003
ABAT	(0.324)	0.723	0.631	0.830	*<0.001*	0.017
IGFBP1	0.315	1.370	1.205	1.558	*<0.001*	0.007
DLGAP5	0.443	1.558	1.284	1.891	*<0.001*	0.035
GGA2	(0.385)	0.680	0.578	0.801	*<0.001*	0.018

SETDB2	(0.330)	0.719	0.624	0.829	*<0.001*	0.024
FAM189A2	(0.377)	0.686	0.585	0.805	*<0.001*	0.018
SFTA3	(0.297)	0.743	0.663	0.833	*<0.001*	0.001
C3orf18	(0.311)	0.733	0.639	0.840	*<0.001*	0.038
TMEM125	(0.317)	0.729	0.638	0.832	*<0.001*	0.014
CLIC6	(0.300)	0.741	0.651	0.844	*<0.001*	0.031
LDHA	0.426	1.531	1.297	1.807	*<0.001*	0.002
C11orf92	(0.341)	0.711	0.616	0.821	*<0.001*	0.016
NKX2-1	(0.272)	0.762	0.682	0.850	*<0.001*	0.005
PLEKHB1	(0.342)	0.710	0.625	0.807	*<0.001*	0.001
IVD	(0.316)	0.729	0.638	0.834	*<0.001*	0.019
TLE1	0.386	1.471	1.268	1.707	*<0.001*	0.002
GNG7	(0.311)	0.733	0.643	0.835	*<0.001*	0.015
CIDEC	0.288	1.334	1.185	1.502	*<0.001*	0.009
GPD1L	(0.320)	0.726	0.632	0.835	*<0.001*	0.033
ARNTL2	0.392	1.480	1.249	1.753	*<0.001*	0.027
FOSL1	0.332	1.394	1.207	1.610	*<0.001*	0.030
PRKCD	(0.306)	0.736	0.648	0.837	*<0.001*	0.014
LOC645166	0.335	1.397	1.207	1.618	*<0.001*	0.035
MGC34034	0.235	1.265	1.147	1.394	*<0.001*	0.011
KIF14	0.406	1.501	1.254	1.796	*<0.001*	0.045
CCT6A	0.299	1.349	1.190	1.528	*<0.001*	0.013
NTSR1	0.272	1.312	1.167	1.476	*<0.001*	0.027
LOC100131726	0.428	1.534	1.286	1.830	*<0.001*	0.009
LYPD3	0.338	1.402	1.217	1.615	*<0.001*	0.013
MYLIP	(0.370)	0.691	0.602	0.793	*<0.001*	0.001
PLCD3	0.331	1.392	1.202	1.613	*<0.001*	0.049
GNMT	(0.395)	0.674	0.573	0.792	*<0.001*	0.008
CXCL17	(0.268)	0.765	0.685	0.855	*<0.001*	0.011
CISH	(0.307)	0.735	0.643	0.841	*<0.001*	0.037
ERO1L	0.392	1.480	1.268	1.728	*<0.001*	0.003
MESDC2	0.296	1.344	1.179	1.533	*<0.001*	0.048
LASS4	(0.366)	0.693	0.602	0.799	*<0.001*	0.002
KLHDC8B	(0.329)	0.720	0.627	0.826	*<0.001*	0.015
ANLN	0.496	1.642	1.360	1.983	*<0.001*	0.001
FUT4	0.334	1.397	1.214	1.606	*<0.001*	0.014
FUT1	(0.364)	0.695	0.595	0.811	*<0.001*	0.020
VAX1	0.303	1.354	1.216	1.507	*<0.001*	0.000
LDLRAD3	0.338	1.402	1.215	1.617	*<0.001*	0.017
SLC47A1	(0.382)	0.683	0.590	0.791	*<0.001*	0.002
ARHGAP11A	0.350	1.420	1.217	1.656	*<0.001*	0.040
C1QTNF6	0.429	1.535	1.296	1.817	*<0.001*	0.003
ITGB1	0.316	1.372	1.195	1.575	*<0.001*	0.035
BZRAP1	(0.395)	0.674	0.583	0.778	*<0.001*	0.000

ABCC6P2	(0.327)	0.721	0.632	0.822	*<0.001*	0.005
SPATA6	(0.304)	0.738	0.651	0.835	*<0.001*	0.008
RGS20	0.363	1.437	1.251	1.651	*<0.001*	0.001
FAM117A	(0.445)	0.641	0.555	0.739	*<0.001*	0.000
EPGN	0.265	1.303	1.159	1.465	*<0.001*	0.045
DENND1C	(0.354)	0.702	0.608	0.809	*<0.001*	0.005
INPP5J	(0.373)	0.689	0.600	0.791	*<0.001*	0.001
PLEK2	0.405	1.500	1.261	1.784	*<0.001*	0.022
KRT18	0.347	1.414	1.214	1.648	*<0.001*	0.040
KRT6B	0.331	1.392	1.211	1.601	*<0.001*	0.016
KRT6C	0.343	1.410	1.230	1.616	*<0.001*	0.004
ZNF540	(0.355)	0.701	0.607	0.809	*<0.001*	0.006
AHSG	0.304	1.356	1.196	1.537	*<0.001*	0.009
SYT10	0.272	1.313	1.165	1.480	*<0.001*	0.038
DTNBP1	(0.365)	0.694	0.599	0.805	*<0.001*	0.006
GNPNAT1	0.462	1.588	1.349	1.868	*<0.001*	0.000
SLC25A42	(0.352)	0.703	0.612	0.808	*<0.001*	0.003
PPP1R13B	(0.314)	0.730	0.637	0.838	*<0.001*	0.033
PKP2	0.354	1.424	1.218	1.665	*<0.001*	0.042
ZNF44	(0.304)	0.738	0.645	0.845	*<0.001*	0.050
GALNT2	0.369	1.446	1.245	1.680	*<0.001*	0.007
GAPDH	0.390	1.477	1.250	1.746	*<0.001*	0.022
LOC441869	(0.360)	0.697	0.608	0.800	*<0.001*	0.001
CREG2	0.298	1.347	1.194	1.520	*<0.001*	0.006
PLK1	0.438	1.549	1.289	1.863	*<0.001*	0.015
KRT6A	0.355	1.426	1.234	1.649	*<0.001*	0.008
IRX5	(0.323)	0.724	0.640	0.819	*<0.001*	0.001
PITX3	0.398	1.489	1.313	1.688	*<0.001*	0.000
VDAC1	0.341	1.407	1.216	1.628	*<0.001*	0.021
RHOV	0.399	1.491	1.273	1.746	*<0.001*	0.004
ZNF77	(0.329)	0.720	0.623	0.832	*<0.001*	0.041
MOCS1	(0.345)	0.708	0.608	0.825	*<0.001*	0.041
FLNC	0.314	1.369	1.194	1.570	*<0.001*	0.033
EXO1	0.405	1.500	1.253	1.795	*<0.001*	0.046
VEGFC	0.304	1.355	1.185	1.550	*<0.001*	0.043
CYP17A1	(0.446)	0.640	0.528	0.776	*<0.001*	0.026
FAM83A	0.563	1.755	1.390	2.217	*<0.001*	0.011
GLS2	(0.341)	0.711	0.619	0.816	*<0.001*	0.006
ESYT3	(0.390)	0.677	0.587	0.781	*<0.001*	0.000
ZFP3	(0.302)	0.739	0.649	0.841	*<0.001*	0.021
ECT2	0.365	1.441	1.226	1.694	*<0.001*	0.046
CMAH	(0.370)	0.691	0.598	0.798	*<0.001*	0.002
DAPK2	(0.377)	0.686	0.580	0.810	*<0.001*	0.045
FKBP4	0.354	1.425	1.228	1.653	*<0.001*	0.015

DKK1	0.384	1.468	1.281	1.682	*<0.001*	0.000
USP4	(0.269)	0.764	0.679	0.861	*<0.001*	0.043
LINGO2	0.325	1.384	1.219	1.571	*<0.001*	0.002
ENPP5	(0.341)	0.711	0.622	0.813	*<0.001*	0.003
IKZF4	(0.317)	0.728	0.633	0.837	*<0.001*	0.038
BTN2A2	(0.313)	0.731	0.640	0.835	*<0.001*	0.018

*HR, hazards ration; LCI, lower limit of confidence interval; UCI, upper limit of confidence interval; FDR, false discovery rate.

Supplementary table 3. 4-gene combination constructed based on LASSO penalty model

**Gene**	**HR**	**LCI**	**UCI**	***P* value**	**Coefficient**
CIDEC	1.334	1.185	1.502	*<0.001*	0.052
ZFP3	0.739	0.649	0.841	*<0.001*	-0.064
DKK1	1.468	1.281	1.682	*<0.001*	0.050
USP4	0.764	0.679	0.861	*<0.001*	-0.084

*HR, hazard ratio; CI, confidence interval; LASSO, least absolute shrinkage and selection operator regression.

Supplementary table 4 Univariate and multivariable Cox proportional hazards regression analysis on the overall survival and recurrence-free survival of patients in the training set, test set, and GSE31210

Datasets and items		OS				RFS			
		Univariate analysis		multivariable analysis		Univariate analysis		multivariable analysis	
		HR (95%CI)	P value	HR (95%CI)	P value	HR (95%CI)	P value	HR (95%CI)	P value
Training set	Risk score	11.962(6.232-22.961)	<0.001	10.559(5.352-20.830)	<0.001	9.281(4.064-21.193)	<0.001	8.028(3.356-19.207)	<0.001
	Age	1.012(0.991-1.034)	0.266	1.019(0.996-1.041)	0.104	0.993(0.971-1.015)	0.535	0.999(0.976-1.023)	0.939
	Gender	1.594(1.074-2.366)	0.021	1.304(0.864-1.969)	0.207	1.130(0.725-1.762)	0.589	1.144(0.727-1.800)	0.56
	Stage	1.214(1.106-1.333)	<0.001	1.158(1.046-1.281)	0.005	1.190(1.059-1.337)	0.003	1.114(0.984-1.262)	0.089
Test set	Risk score	5.377(1.736-16.657)	0.004	7.168(2.330-22.058)	0.001	2.949(0.800-10.867)	0.104	5.668(1.400-22.942)	0.015
	Age	1.000(0.978-1.023)	0.991	1.006(0.984-1.028)	0.616	1.028(1.002-1.054)	0.036	1.031(1.005-1.058)	0.017
	Gender	0.716(0.461-1.113)	0.138	0.553(0.347-0.881)	0.013	0.786(0.489-1.264)	0.321	0.642(0.383-1.078)	0.094
	Stage	1.313(1.192-1.446)	<0.001	1.342(1.206-1.493)	<0.001	1.167(1.043-1.307)	0.007	1.196(1.055-1.356)	0.005
GSE31210	Risk score	1.047(1.016-1.079)	0.003	1.029(0.997-1.062)	0.076	1.042(1.018-1.066)	<0.001	1.024(1.000-1.049)	0.052
	Age	1.025(0.977-1.075)	0.306	1.027(0.981-1.076)	0.257	1.034(0.997-1.072)	0.074	1.035(0.999-1.073)	0.059
	Gender	1.519(0.780-2.955)	0.219	0.922(0.355-2.396)	0.867	1.271(0.778-2.075)	0.338	0.853(0.425-1.712)	0.655
	Smoking	0.611(0.312-1.195)	0.15	0.756(0.291-1.964)	0.565	0.750(0.459-1.227)	0.252	0.830(0.416-1.655)	0.596
	Stage	2.030(1.486-2.775)	<0.001	1.883(1.353-2.621)	<0.001	1.817(1.450-2.278)	<0.001	1.723(1.352-2.194)	<0.001

Abbreviations: OS, overall survival; RFS, recurrence-free survival

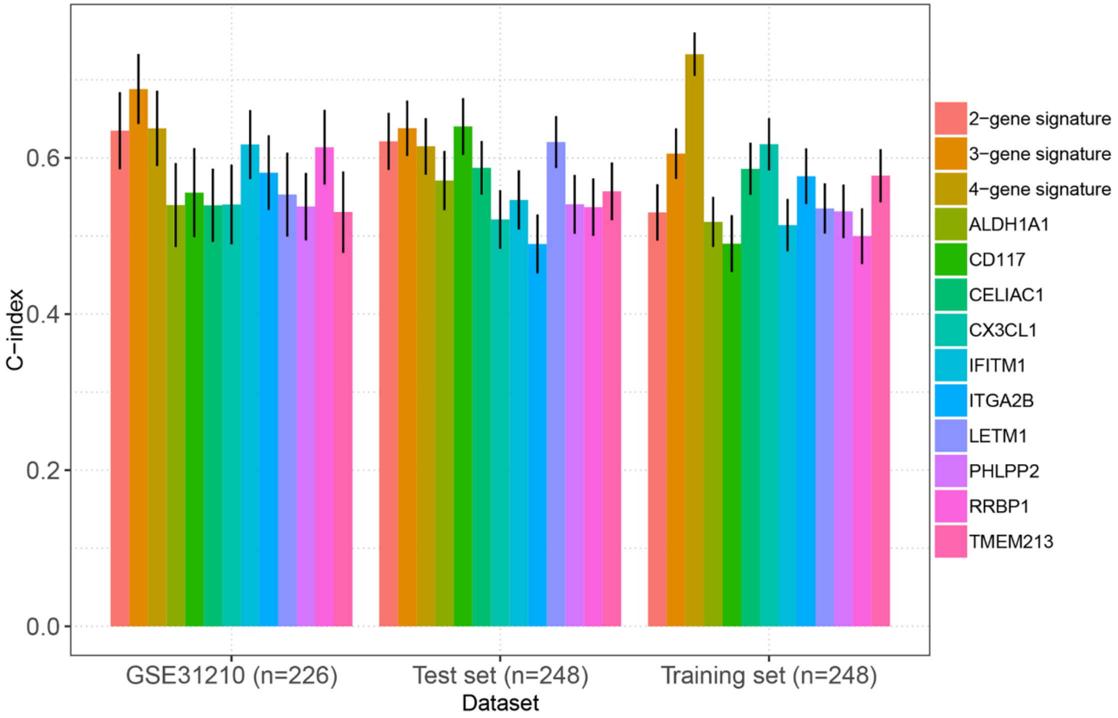

Supplementary figure 1 Comparison of the prognostication performance between the 4-gene combination with other biomarkers in lung adenocarcinoma
